# Bovine Highly Pathogenic Avian Influenza Virus Stability and Inactivation in the Milk Byproduct Lactose

**DOI:** 10.3390/v16091451

**Published:** 2024-09-12

**Authors:** Taeyong Kwon, Jordan T. Gebhardt, Eu Lim Lyoo, Mohammed Nooruzzaman, Natasha N. Gaudreault, Igor Morozov, Diego G. Diel, Juergen A. Richt

**Affiliations:** 1 Department of Diagnostic Medicine/Pathobiology, College of Veterinary Medicine, Kansas State University, Manhattan, KS 66506, USA; 2 Department of Population Medicine and Diagnostic Sciences, College of Veterinary Medicine, Cornell University, Ithaca, NY 14853, USA

**Keywords:** lactose, highly pathogenic avian influenza virus, stability, citric acid inactivation, heat inactivation

## Abstract

The recent incursion of highly pathogenic influenza viruses into dairy cattle opens new insights for influenza virus ecology and its interspecies transmission and may have a significant impact on public health and agriculture. The aim of this study was to determine the stability of a bovine highly pathogenic avian influenza H5N1 virus isolate in the milk byproduct lactose and to evaluate two inactivation methods using industrial procedures. The bovine isolate of the highly pathogenic avian influenza H5N1 virus was stable for 14 days in a concentrated lactose solution under refrigerated conditions. Heat or citric acid treatments successfully inactivated the virus in lactose. This study highlights the persistence of HPAIV in lactose and its efficient inactivation under industrial standards.

## 1. Introduction

Recently, clade 2.3.4.4b viruses were detected in dairy cattle populations in the United States [1,2]. Infected cattle exhibited reduced appetite, fever, mild respiratory symptoms, reduced milk production, and changes in milk quality [3,4]. High levels of virus shedding in milk have been detected in affected cows, with virus titers ranging from 10^4^ to 10^8.8^ TCID_50_/mL [3]. In contrast, an experimental infection of calves with a bovine H5N1 isolate via intra-nasal and oral routes resulted in moderate virus shedding with no obvious clinical signs [4]. It is presumed that a single introduction from wild birds to cattle occurred, and subsequently, the movement of subclinical cattle played a significant role in its spread to multiple sites [5]. Four human infections with HPAI H5N1 viruses following exposure to dairy cattle have been reported so far [6,7]. Given the high titer of the virus in milk and the potential for H5N1 transmission via raw milk and its byproducts to humans and agricultural animals, it is essential to develop appropriate processes to inactivate the bovine H5N1 virus in these substrates to mitigate the risk of transmission. Current knowledge and techniques have focused on the pasteurization of milk, which is widespread within the dairy industry and has been shown to be effective for HPAI viruses [8,9,10]. The consumption of contaminated materials is presumably considered a major route of HPAI infection in pet and wild mammals [11]. Therefore, additional research is needed to validate inactivation processes in milk and its byproducts, such as dried whey, whey permeate, and lactose, which are used for animal nutrition in agriculture. Therefore, this study aimed to determine the stability of a bovine H5N1 isolate in the milk byproduct lactose and to evaluate two inactivation methods using industrial procedures.

## 2. Materials and Methods

The bovine isolate of HPAI H5N1 clade 2.3.4.4b, isolate A/Cattle/Texas/063224-24-1/2024 [3], was propagated and titrated on MDCK cells. The virus stock was mixed with a concentrated lactose solution at 1:10 dilution, and 1 mL of contaminated lactose was incubated at refrigerated temperatures (4.4 °C to 5.6 °C) and titrated on MDCK cells.

For inactivation, virus-spiked lactose at a 1:10 dilution was subjected to heat or citric acid treatments. A total of 1 mL of the H5N1-spiked lactose was incubated at 63, 66, or 99 °C for up to 30 min and cooled down on ice water for at least 30 min. The temp 63 °C for 30 min was chosen since it is used by the food industry for pasteurization. Other temperatures were chosen to evaluate the efficacy of elevated temperatures and shorter time periods. For citric acid treatment, 1 mL of the H5N1-spiked lactose was mixed with 1 mL of different levels of citric acid and incubated at refrigerated temperatures (4.4 °C to 5.6 °C). After a defined incubation time, the sample was neutralized by adding 1N NaOH. All samples were titrated on MDCK cells, and the presence of infectious viruses were visualized by an immunofluorescence assay.

## 3. Results

**Figure 1:**
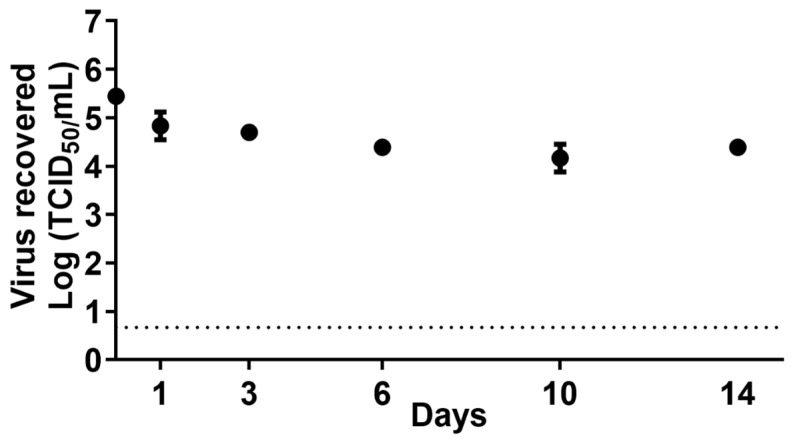
The stability of the bovine isolate of HPAI H5N1 clade 2.3.4.4b in a concentrated lactose solution. The virus was mixed with whole milk and lactose at 1:10 dilution and incubated at a refrigerated temperature. At each time point, the samples were titrated on MDCK cells. Dash line represents the limit of detection, 4.64 TCID_50_/mL.

The bovine H5N1 virus was rather stable in lactose at a refrigerated temperature, and there was only a 1-log reduction after incubation for 14 days (Figure 1). Heat treatment at 63 °C for 5 min resulted in 3-log reduction in both high- and low-dose samples, and all samples were virus-negative at 15 and 30 min (Table 1). No infectious virus was isolated after heat treatment at 66 °C and 99 °C for a minimum of 5 min. Next, we investigated the effect of citric acid treatment on virus inactivation in the concentrated lactose solution. In high-dose samples, low levels of the virus were still present after treatment with 0.2% and 0.4% citric acid for up to 1 h, but the virus was inactivated after 0.6% citric acid treatment. For low-dose samples, 0.2% and 0.4% citric acid treatment successfully inactivated H5N1 within 20 min, and 0.1% treatment was effective at inactivating the virus after 40 min contact time (Table 1).

## 4. Discussion

Our findings highlight bovine H5N1 virus survival in concentrated lactose solution for up to 14 days at holding temperature and emphasize its successful inactivation by pasteurization (63 °C) and citric acid treatment. In this study, samples with high virus titers are consistent with the observed range of virus titers in milk from infected cows [3,4], and the low-titer samples would theoretically be possible if milk from infected animals is diluted with milk from non-infected animals. Thus, the viral titers used in the current experiment are representative of possible virus levels in milk and its coproducts. In previous studies, heat treatment at 72 °C for 15 s completely inactivated the virus in spiked raw milk collected from healthy dairy cattle [12], but the virus was still viable after 72 °C for 30 s in H5-positive milk from infected animals [9]. Although the present study evaluated the inactivation of the virus in the spiked samples, experiments using milk byproducts derived from H5N1-contaminated milk would provide additional insights into the efficacy of inactivation procedures of milk byproducts.

This is the first study to investigate the inactivation of a bovine HPAI H5N1 virus in the milk coproduct, concentrated lactose, which is frequently used as a feed ingredient for agricultural animals including pigs as well as for other purposes. In summary, H5N1-contaminated milk byproducts might pose a risk to animal health if consumed untreated. This study provides insights on the persistence of bovine HPAIV in dairy byproducts and effective inactivation strategies under industrial standards.

## Figures and Tables

**Table 1:** Inactivation of the bovine isolate of HPAI H5N1 clade 2.3.4.4b in a concentrated lactose solution.

Treatment	Virus Dose	Condition	Time and Virus Titer ^1^ (TCID_50_/mL)			
			0 min	5 min	15 min	30 min
Heat	High	63 °C	3.59 × 10^5^	4.64 × 10^2^	Negative	Negative
		66 °C		Negative	Negative	Negative
		99 °C		Negative	Negative	Negative
	Low	63 °C	1.29 × 10^3^	3.78 × 10^0^	Negative	Negative
		**Acidulant level**	**0 min**	**20 min**	**40 min**	**60 min**
Citric acid	High	0.2%	2.15 × 10^5^	7.74 × 10^0^	5.99 × 10^0^	6.14 × 10^0^
		0.4%		3.78 × 10^0^	3.68 × 10^0^	4.64 × 10^0^
		0.6%		Negative	Negative	Negative
	Low	Untreated	1.29 × 10^3^	1.00 × 10^3^	1.29 × 10^3^	1.29 × 10^3^
		0.1%		3.68 × 10^0^	Negative	Negative
		0.2%		Negative	Negative	Negative
		0.4%		Negative	Negative	Negative

^1^ The limit of detection of this assay was 4.64 TCID_50_/mL.

## Data Availability

Raw data are available upon request.
